# Maternal and infant outcomes of pregnancy associated with anti-SSA/RO antibodies: a systematic review and meta-analysis

**DOI:** 10.1186/s12969-023-00803-0

**Published:** 2023-03-04

**Authors:** Xiangrui Sheng, Xiaohui Song, Yue Xiong, Tian Ren, Xin Chang, Jian Wu, Jing Cao, Tao Cheng, Mingjun Wang

**Affiliations:** Department of Rheumatology, The First Affiliated Hospital of Soochow University, No.188 Shizi St, Suzhou, 215006 Jiangsu China

**Keywords:** Anti-SSA/RO, NLE, CHB, Pregnancy

## Abstract

**Objective:**

The relationship between anti-SSA/RO antibodies and pregnancy has been reported previously, and we aim to visualize the rates of maternal and infant outcomes with anti-SSA/RO.

**Methods:**

We systematically searched records from Pubmed, Cochrane, Embase, and Web of Science databases, pooled incidence rates of adverse outcomes of pregnancy, and 95% confidence intervals (CIs) were performed with RStudio.

**Results:**

A total of 890 records comprising 1675 patients and 1920 pregnancies were searched from the electronic databases. For maternal outcomes, the pooled estimate rates were 4% for termination of pregnancy, 5% for spontaneous abortion, 26% for preterm labor, and 50% for cesarean operation. While for fetal outcomes, the pooled estimate rates were 4% for perinatal death, 3% for intrauterine growth retardation, 6% for endocardial fibroelastosis, 6% for dilated cardiomyopathy, 7% for congenital heart block, 12% for congenital heart block recurrence, 19% for cutaneous neonatal lupus erythematosus, 12% for hepatobiliary disease and 16% for hematological manifestations. A subgroup analysis of congenital heart block prevalence was performed, diagnostic method and study region were found to affect heterogeneity to some extent.

**Conclusion:**

Cumulative analysis of data from real-world studies confirmed adverse pregnancy outcomes of women with anti-SSA/RO, serves as a reference and a guide for the diagnosis and subsequent treatment of these women, thereby enhancing maternal and infant health. Additional studies with real-world cohorts are required to validate these results.

**Supplementary Information:**

The online version contains supplementary material available at 10.1186/s12969-023-00803-0.

## Introduction

Anti-SSA/RO, a crucial component of serological markers, is frequently used to diagnose Sjogren’s syndrome and Systemic lupus erythematosus (SLE). The management of pregnancy in women with autoimmune diseases is undoubtedly an integral part of the treatment of the disease. Neonatal Lupus Syndrome (NLE) is a condition associated with anti-SSA/RO antibodies that affect the fetal myocardium during pregnancy and can lead to dilated cardiomyopathy, endocardial fibroelastosis (EFE), congenital heart block (CHB), and skin, liver, and hematological system [1]. The prevailing theory is that maternal anti-SSA/RO antibodies are deposited in the fetal myocardial tissue via passive placental transmission, resulting in subsequent inflammation and fibrosis [2, 3].

In addition to the CHB mouse model constructed by Carús et al. [4], pathological examination of the hearts of 10 children who died of EFE by Nield [5] combined with relevant clinical studies [6] has further validated the association of maternal antibodies with poor pregnancy outcomes. Furthermore， high titers of maternal antibodies were found to be associated with fetal damage in a study that was conducted prospectively by Edgar Jaeggi et al. [7].

However, most existing clinical studies have restrictions such as insufficient participants, narrow scopes, and limited endpoints. Given these premises, we aimed to systematically estimate the rates of maternal and infant outcomes of pregnant women with anti-SSA/RO antibodies.

## Materials and methods

### Study design

This study adheres to the Preferred Reporting Items for Systematic Review and Meta-Analysis Protocols (PRISMA-P) statement. This review is registered on PROSPERO (CRD42022384043).

### Search strategy

We searched Pubmed, Cochrane, Embase, and Web of Science databases for records up to October 2022, using the terms “anti-RO” or “anti-SSA” and the following combinations “pregnancy” “Pregnant Women” “mothers” “maternal” and “fetal”.

### Inclusion and exclusion criteria

Studies that meet the following criteria were included:

- A real-world observational study of pregnancy outcomes in anti-SSA/RO-positive pregnant women.
- They provided research methods and specific data.
- They were published in 2000 or later.

Studies that meet the following criteria were excluded:

- Reviews, case reports, or conference abstracts.
- Articles not in English.
- Articles for which full text was not available were excluded.

### Outcomes analyzed

The following maternal endpoints were analyzed: termination of pregnancy, spontaneous abortion, preterm labor, and cesarean operation. The following fetal outcomes were analyzed: perinatal death, intrauterine growth retardation, endocardial fibroelastosis (EFE), dilated cardiomyopathy (DCM), congenital heart block (CHB), congenital heart block recurrence and cutaneous neonatal lupus erythematosus (CNLE).

### Data collection and quality assessment

Initial screening was based on titles and abstracts, followed by a full-text reading of studies that met inclusion criteria and ultimate exclusion based on exclusion criteria. Information from the included literature was documented in a specific database, including author, publication date, study population, study type, study region, enrollment time, sample size, number of positives, etc. The Newcastle-Ottawa Scale (NOS), which includes three components: choice of study groups, comparability of cohort and control groups, and determination of outcome, was used to assess the quality of included studies. Three or fewer were regarded as low, four to six as intermediate, and nine or more as an outstanding quality. The aforementioned work was completed independently by 3 authors (XRS, XHS, and YX), and disputes were discussed.

### Statistical analysis

Statistical analysis was performed with RStudio (version 2022.07.2) and Review Manager (version 5.4). For non-normally distributed incidence rates, logit transformation was used to make them close to a normal distribution, and normality tests were performed by using the Shapiro-Wilk test. Pooled incidence rates of outcomes and 95% confidence intervals (CIs), were calculated by the random-effect model or fix-effect model. Furthermore, heterogeneity between studies was assessed using I^2^. Sources of heterogeneity were explored through subgroup analysis. Sensitivity analysis was conducted by omitting each study sequentially to evaluate the stability of the study results.

## Results

### Searched outcomes

**Fig. 1:**
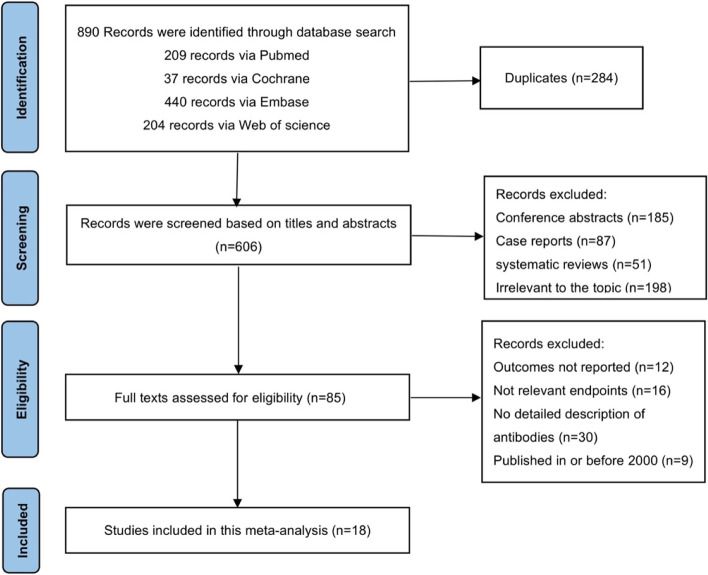
Flow diagram representing the study selection

A total of 890 records were searched from the above-mentioned electronic databases. Two hundred eighty-four duplicate records were removed. Five hundred twenty-one studies were screened based on the titles and abstracts. Sixty-seven studies were excluded after reading the full texts. Ultimately, 18 articles that met the screening criteria were included in this meta-analysis as shown in Fig. 1.

#### Study characteristics

The main characteristics of the included studies were shown in Table 1, including study type, study region, enrollment period, and the number of participants. This analysis encompassed a total number of 1675 patients and 1920 pregnancies. And they were enrolled from the year 1987–2019 and distributed in different regions such as Canada, France, Italy, Sweden, and the USA. As no RCT trials were included in our meta-analysis, we carried out a quality assessment by using the Newcastle-Ottawa scale (NOS). The NOS assessment results for the 20 studies were 5–8.

**Table 1:** Main characteristics of the studies included in this analysis

Study (year)	Country	Study design	Period(y)	Participants (pregnancies)	Maternal outcomes	Fetal outcomes	NOS score
Ruffatti 2016 [8]	Italy	retrospective	2009–2014	12(12)	type of delivery, pregnancy complications, gestational period (weeks), preterm labor	5 min APGAR score, neonatal complications, birth weight, cardiac complications, NICU admission, small for gestational age	******
Zuppa 2008 [9]	Italy	retrospective	2004–2005	10(10)	type of delivery, gestational period (weeks), preterm labor	live birth, birth weight, hematologic manifestation, echoencephalographic abnormalities, liver manifestations	******
Morel 2017 [10]	France	–	2000–2014	174(187)	gestational period (weeks), preterm labor	fetal death /perinatal death, birth weight, DCM, EFE, hydrops, Pericardial effusion, congenital heart disease, pacemaker implantation	*****
Cimaz 2003 [11]	Canada	prospective	1987–2000	112(128)	preterm labor	live birth, type of block, skin manifestations, hematologic manifestation, liver manifestations, prolonged QT interval, CHB recurrence, hematological and hepatic manifestations	******
Boros 2007 [12]	Canada	–	1999–2004	75(87)	–	type of block, hydrocephalus, cutaneous involvement, hematologic and/or liver enzyme abnormalities, hematological and hepatic manifestations	******
Miniaoui 2022 [13]	France	–	2000–2019	215(215)	terminations of pregnancy, disease-free survival probability at 5 years and 10 years, the probability of developing SLE/SS, the estimated median time to progression	fetal death /perinatal death, live birth	******
Gladman 2002 [14]	Canada	prospective	1988–1997	105(118)	spontaneous abortion	intrauterine deaths, DCM, type of block, peripheral pulmonary artery stenosis, CHB recurrence	******
Pisoni 2010 [15]	Italy	prospective	2004–2008	22(24)	terminations of pregnancy	type of block, cutaneous involvement, CHB recurrence	******
Skog 2015 [16]	Sweden	prospective	2000–2013	155(212)	type of delivery, preterm labor, preeclampsia	fetal death /perinatal death, type of block, CHB recurrence, neonatal complications	*****
Jaeggi 2010 [7]	Canada	prospective	2000–2008	164(186)	termination of pregnancy	intrauterine death, type of block, endocardial fibroelastosis, hydrops, pericardial effusion, CHB recurrence, hematological and hepatic manifestations	******
Sonesson 2004 [17]	Sweden	–	1999–2003	24(24)	preterm labor	type of block	********
Kan 2017 [18]	Canada	retrospective	2009–2014	189(195)	maternal eclampsia	intrauterine death, pacemaker implantation, type of block, endocardial fibroelastosis, pericardial effusion, congenital heart disease, CHB recurrence	******
Bergman 2012 [19]	Sweden	crosssectional follow-up	1999–2007	48(57)	–	type of block	*****
Gerosa 2007 [20]	Italy	prospective	2000–2004	60(60)	spontaneous abortion, preterm labor	pacemaker implantation,1 min APGAR score, type of block	********
Motta 2007 [21]	Italy	prospective	1999–2005	43(51)	–	type of block	******
Chalumeau 2004 [22]	France	–	1991–2002	106(167)	spontaneous abortion, terminations of pregnancy, preterm labor	IUGR，fetal death /perinatal death，live birth，type of block, congenital defects	********
Brucato 2002 [23]	Italy	prospective	–	100(126)	type of delivery, ectopic pregnancy, spontaneous abortion, preeclampsia, pregnancy complications, preterm labor	live birth, small for gestational age, type of block, IUGR	********
Briassouli 2013 [24]	USA	retrospective	–	61(61)	type of delivery	–	*****

### Maternal outcomes

**Fig. 2:**
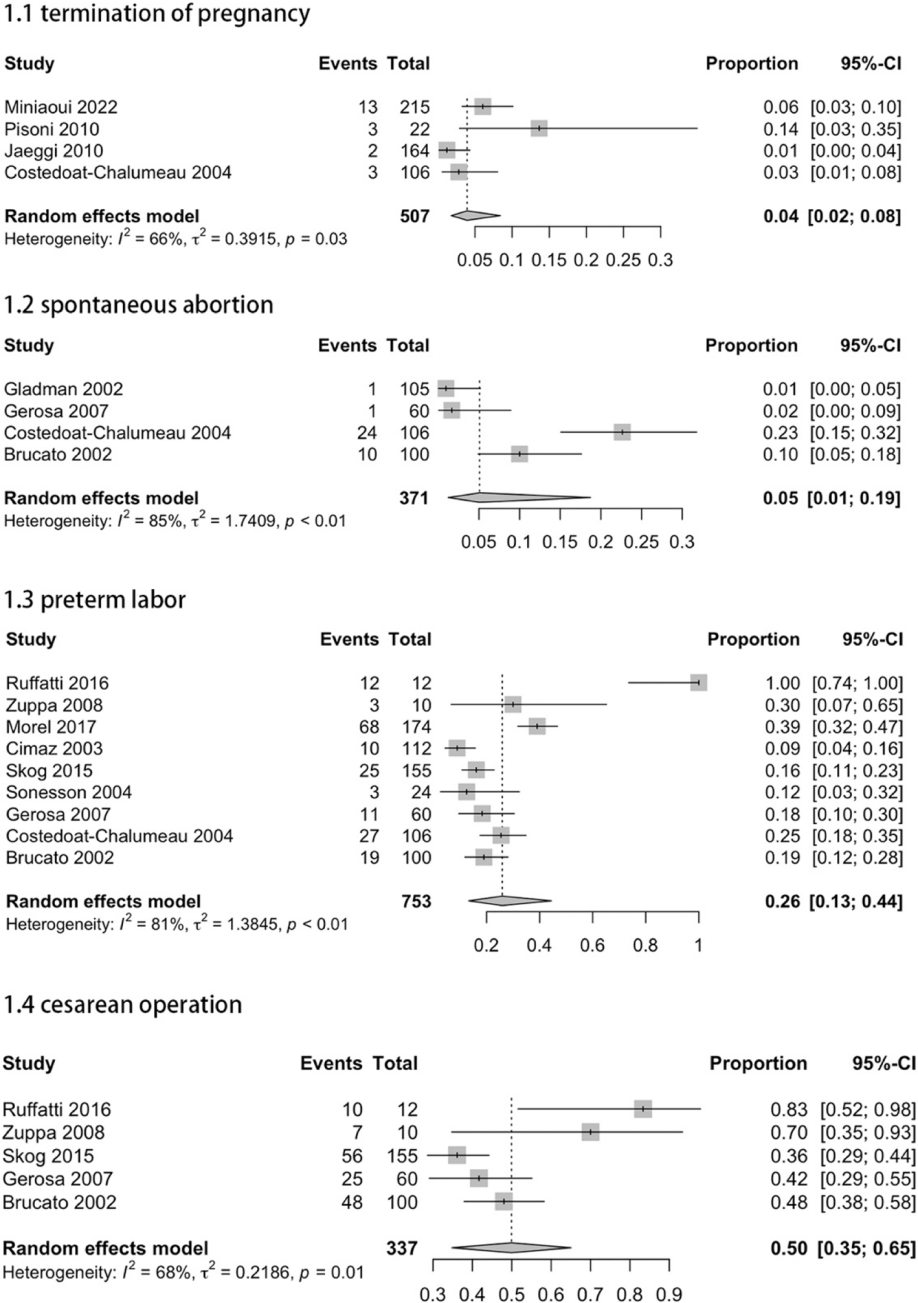
Adverse maternal outcomes in pregnant women with anti-SSA/RO

Regarding all 4 studies reporting termination of pregnancy data of women with anti-SSA/Ro positive, the pooled estimate rate for this outcome was 4% (95% CI: 2–8%; I^2^ = 66%). The 4 studies reporting spontaneous abortion yielded an incidence rate of 5% (95% CI: 1–19%; I^2^ = 85%). For preterm labor, the combined 9 studies reported an incidence of 26% (95% CI: 13–44%; I^2^ = 81%). Combining the data from the 5 studies on cesarean operation, the pooled estimate rate was 50% (95% CI: 35–65%; I^2^ = 68%) (Fig. 2).

### Fetal outcomes

Meta-analyses of pooled study data demonstrated that the stillbirth or perinatal death rate was 4% (95% CI: 2–6%; I^2^ = 76%). Four studies reported IUGR outcomes in pregnant women exposed to anti-SSA/RO antibodies and the pooled data was 3% (95% CI: 0–6%; I^2^ = 71%). The analysis results showed that the incidence of Endocardial fibroelastosis was 6% (95% CI: 2–17%; I^2^ = 92%).

**Fig. 3:**
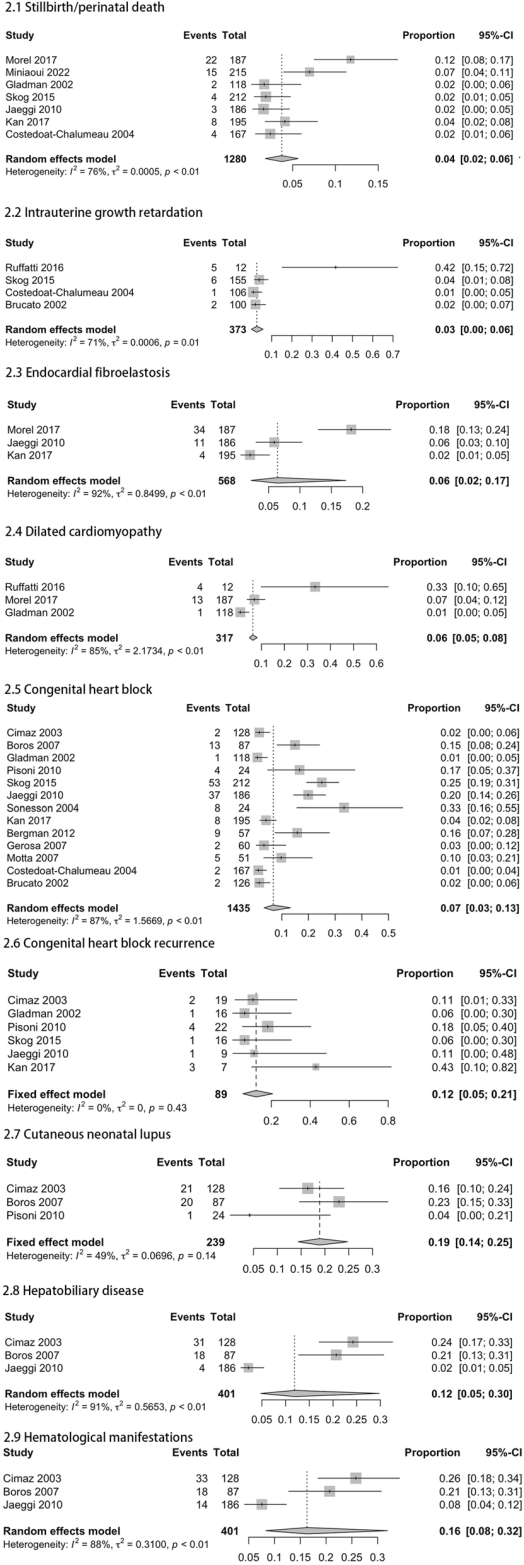
Adverse fetal outcomes observed in pregnant women with anti-SSA/RO

Concerning dilated heart disease, the combined 3 reports reported this incidence to be 6% (95% CI: 5–8%; I^2^ = 85%). Regarding the incidence of CHB, the combined 13 studies reported an incidence of 7% (95% CI: 3–13%; I^2^ = 87%). There was no difference in the prevalence of CHB between SLE and non-SLE groups based on the mother’s diagnosis (Fig. S1). The 6 studies reporting the recurrence of CHB yielded a recurrence rate of 12% (95% CI: 5–21%; I^2^ = 0%). Combining the 3 studies on CNLE data, the pooled estimate rate was 19% (95% CI: 14–25%; I^2^ = 49%). The pooled estimate rate of hepatobiliary disease was 12% (95% CI: 5–30%; I^2^ = 91%) and the pooled estimate rate of hematological manifestations was 16% (95% CI: 8–32%; I^2^ = 88%) (Fig. 3).

### Subgroup analysis

Due to the large heterogeneity of study outcomes in this paper and given the small number of articles included for the remaining outcomes, we further performed a subgroup analysis of CHB prevalence, grouped according to study region, publication time, and diagnostic method. By calculating I^2^, we could conclude that all of them (Figs. S2, S3 and S4) affected heterogeneity to some extent.

### Sensitivity analysis and publication bias analysis

We conducted sensitivity analysis by omitting each study sequentially and no significant changes were observed, indicating that the results of the meta-analysis were relatively stable (Fig. S5). As most of the studies included in this paper did not have a control group and were mainly observational studies on prevalence outcomes with no or little publication bias, no correlation analysis was conducted.

## Discussion

In this article, we studied the incidence of various pregnancy outcomes for both mother and baby in anti-SSA/RO-positive pregnant women. Stillbirth and perinatal mortality both occurred in 4% of pregnancies, whereas intrauterine growth restriction occurred in 6% of pregnancies, pregnancy termination occurred in 4% of pregnancies, and 5% of pregnancies ended in spontaneous abortion. CHB is the major cause of these unfavorable pregnancies, and our findings show a prevalence of 7% of anti-SSA/RO-associated CHB. Previous prospective studies in women with anti-SSA/RO antibodies have shown a risk of cardiac NL of approximately 2% if the mother didn’t have a previously affected pregnancy [11, 25, 26]. However, in the study by Buyon [27], mothers with a previous CHB pregnancy outcome were two to three times more likely to have another pregnancy that remained CHB than those mothers with anti-SSA/RO antibodies who had never had an affected child, which may explain the difference in our analysis. A further pooling of data from six articles suggested a CHB recurrence rate of 12% would further support this conclusion.

A large US cohort study [28] showed that maternal health status, steroid regimen, antibody level, and disease severity of the first affected child did not predict recurrence, and the relatively high recurrence rate suggests that mothers with previously affected children should be targeted for monitoring and treatment. In addition to focusing on the subsequent births of these particular mothers, given the familial aggregation of autoimmune diseases [29], we need to further assess the future health of those siblings of children with CHB who are currently temporarily healthy. In their study, Johannes Mofors and colleagues evaluated the risk of disease in the siblings of children diagnosed with CHB [30]. They discovered that these children’s siblings had an increased risk of autoimmune disease; however, the authors concluded that this finding may be biased because siblings of patients diagnosed with chronic CHB may undergo medical screening more frequently than the general population.

We also analyzed the prevalence of CHB according to whether the mother had SLE or not. After integrating the data from three studies, there was no significant difference between the two groups, suggesting that the pregnancy outcome of mothers with positive anti-SSA/RO antibodies was not related to the type of autoimmune disease, but closely related to their antibodies. Miniaoui [13] followed 157 asymptomatic mothers for 11 years and found that the probability of asymptomatic mothers developing SLE was 12.7% and that of asymptomatic mothers developing SS was 28.1%.

We discovered skin lesions in roughly 19% of the babies born to moms with antibody positivity. In contrast to cardiac NLE, CNLE most commonly manifests as a transient, reversible cutaneous lesion that is analogous to subacute cutaneous lupus erythematosus. The rash most commonly appears in the periorbital and spectacle-like areas of the face. However, in some patients, it can also be seen in the vulvar triangle and on the soles of the feet as erythematous dusky patches [31]. The skin lesions often emerge for the first time at the age of 12 weeks and last until the age of 6 months [1], which is roughly the same time as autoantibodies are eliminated from the infant’s serum. According to the findings of a study conducted by Izmirly, fetuses who have had previous cutaneous lesions are at a higher risk of future recurrent NLE, in particular cardiac lesions, somewhere between 6 and 10 times more frequently than other children without previous affected conditions [32]. In addition, we found that 16% of the infants developed hematological abnormalities mainly characterized by three-line reduction, and 12% of the infants developed liver diseases mainly characterized by liver enzyme abnormalities. These abnormalities mostly disappeared by the first year of life, with no clinical manifestations.

The antibody-mediated myocardial injury usually progresses gradually, starting with first-degree AVB to irreversible second and third-degree AVB, and possibly even EFE and DCM. The latter two both had an incidence of about 6% according to our analysis. Given the high mortality rate of these two diseases [33], most fetuses may be born dead. Considering the small number of studies we included and the fact that echocardiography is a highly subjective diagnostic modality, the data require further validation.

However, no similar cases have been reported in the maternal heart, and we believe that this antibody damage is unique to the fetal stage of development. Considering such a low incidence, there is evidence that the pathogenesis may involve more than antibodies derived from mothers. In recent years hypotheses have also emerged regarding cumulative genetic transmission [34], fetal MHC [35, 36] cardiac manifestations of allograft rejection and graft-versus-host disease [37], Type-I IFN system activation [38], and the protective role of β2-GPI has also emerged [39].

Currently, routine antenatal testing does not detect autoimmune antibodies, and the majority of women who are positive for anti-SSA/RO antibodies have no clinical manifestations.

Most CHB cannot be diagnosed early and 70% of them die in utero [40]. Among live births, the average 10-year survival rate is 86% [3]. In current clinical practice, most fetal screening starts at 13–16 weeks. However, a review by Norbert Gleicher [6] suggests that the associated pathological changes begin early in the second trimester. This reminds us that anti-SSA/RO antibodies are IgG. From the 13th week [41], IgG antibodies are transferred from the mother to the fetus via the placenta, and combined with the timing of the formation of the anatomical function of the cardiac conduction system, damage to cardiac conduction tissue occurs as early as approximately 8–13 weeks of gestational age [42]. It is very crucial to start prenatal monitoring. Previously, abnormalities were primarily detected during a routine fetal ultrasound. However, Carolina Llanos et al. [43] found that some histologically definite EFE were not detected on echocardiography after the autopsy of 18 antibody-positive fetuses, and therefore fetal echocardiography may not be a sensitive enough diagnostic tool. The study has shown [19] that some infants with no cardiac manifestations before or after birth were diagnosed with CHB during follow-up, which may also serve as a side-effect validation of the limitations of the available diagnostic tools.

Common clinical treatments currently available include hydroxychloroquine, steroids, plasmapheresis, and intravenous gamma globulin (IV-Ig). Our study found that in maternal outcomes, the incidence of preterm birth reached a staggering 26%. With regard to treatment in terms of preterm birth, we found that in a retrospective cohort study [44], the rate of preterm birth was significantly lower in SLE pregnant women exposed to hydroxychloroquine than in those not exposed. Although this study was limited to patients with a diagnosis of SLE, the results also suggest some protective effect of hydroxychloroquine in the treatment of preterm labor, considering that 40% of SLE patients have anti-SSA/RO antibodies [3]. Among the above treatment regimens, which is more effective in reducing the rate of preterm birth and which is more effective in improving maternal antibody titers among different treatments is still unknown. Moreover, this paper did not stratify according to antibody titers, so further relevant studies are needed in the future to achieve more targeted prevention.

This is the first meta-analysis of maternal and infant outcomes in women with anti-SSA/RO antibodies. The results of our subgroup analysis suggested that diagnostic methods, regions of studies, and study period might be sources of heterogeneity. Because some studies employed only one certain diagnostic method and others used both echocardiography and ECG as screening tools, which we believe influenced disease detection rates to some extent. Echocardiography is a highly subjective diagnostic modality that is influenced by the physician’s experience and expertise so regional differences and publication time could also affect the diagnosis of the disease. In addition, we believe the following factors may also affect heterogeneity: 1. The inclusion of patients with varying treatment regimens may affect the results of the analysis; 2. Medication adherence, follow-up time, etc. Consequently, subgroup analysis may not adequately account for all the heterogeneity. Due to the absence of a control group, a meta-analysis of pooled prevalence typically has greater heterogeneity. All the literature used in this study met the inclusion criteria and the data from each study conformed to a normal distribution. Despite the greater heterogeneity, it is hoped that the combined data in this paper will be available to all researchers.

## Conclusion

In conclusion, this meta-analysis further clarifies the impact of anti-SSA/RO antibodies on maternal and infant outcomes during pregnancy. Therefore, it serves as a reference and a guide for the diagnosis and subsequent treatment of these women, thereby enhancing maternal and infant health. Additional studies with real-world cohorts are required to validate these results.

## Supplementary Information

**Additional file 1: Fig. S1.** The prevalence of CHB between SLE and non-SLE groups. **Fig. S2.** Subgroup analysis of different types of diagnostic method. **Fig. S3.** Subgroup analysis based on regions of studies. **Fig. S4.** Subgroup analysis based on publication time. **Fig. S5.** Sensitivity analysis.

## Data Availability

The datasets used and/or analyzed during the current study are available from the corresponding author on reasonable request.

## Abbreviations

NLE: Neonatal lupus syndrome
EFE: Endocardial fibroelastosis
CHB: Congenital heart block
DCM: Dilated cardiomyopathy
CNLE: Cutaneous neonatal lupus erythematosus
