# Supplementary material for: Maternal and infant outcomes of pregnancy associated with anti-SSA/RO antibodies: a systematic review and meta-analysis
Source: Pediatr Rheumatol Online J. 2023 Mar 4;21:22. doi: 10.1186/s12969-023-00803-0 (PMC9985242; doi:10.1186/s12969-023-00803-0)

**Supplements**

Fig.S1 the prevalence of CHB between SLE and non-SLE groups


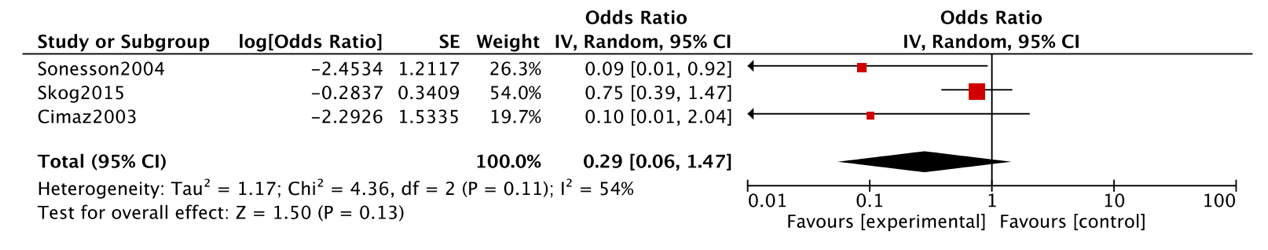


Fig.S2 subgroup analysis of different types of diagnostic method


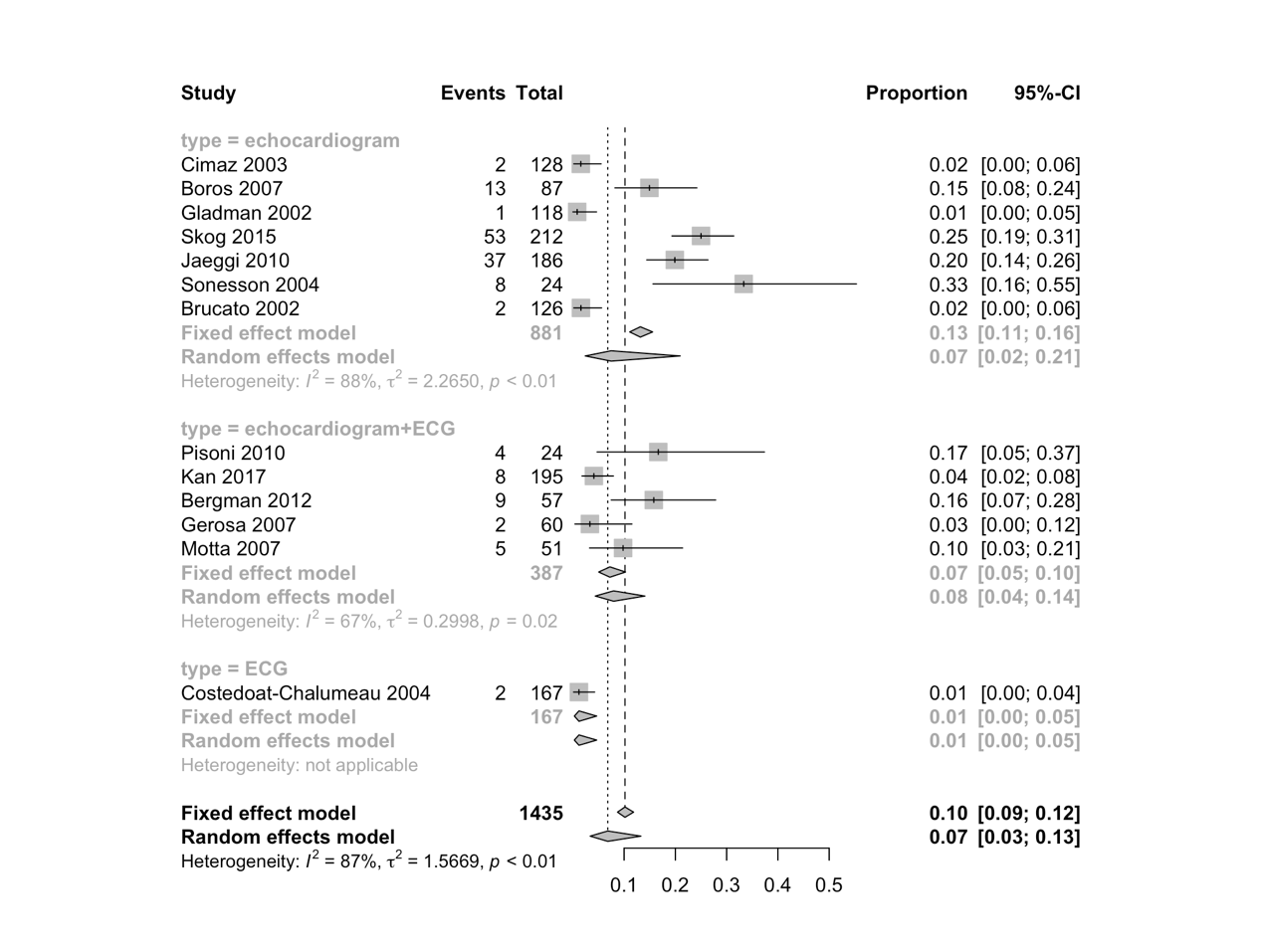


Fig.S3 subgroup analysis based on regions of studies


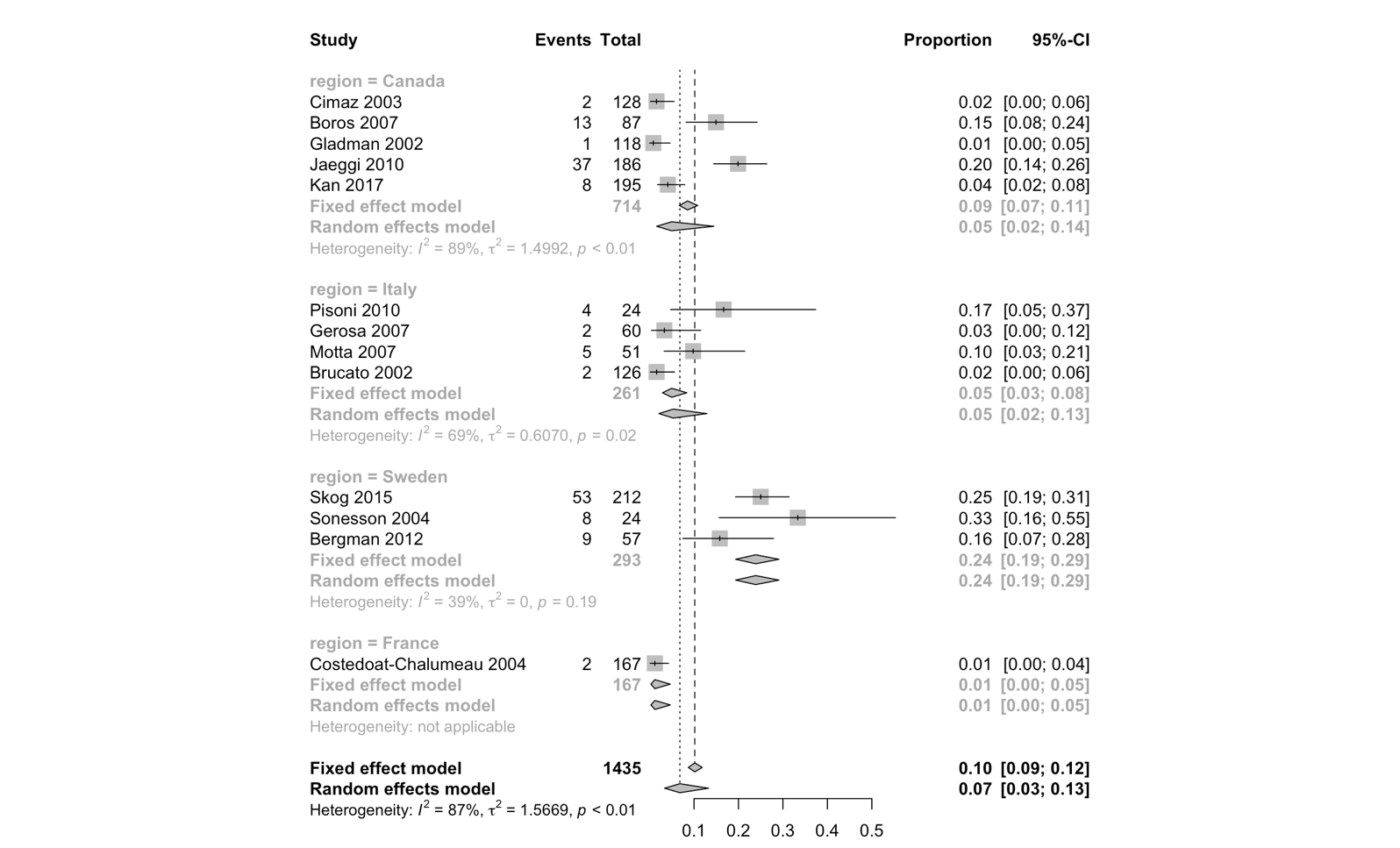


Fig.S4 subgroup analysis based on publication time


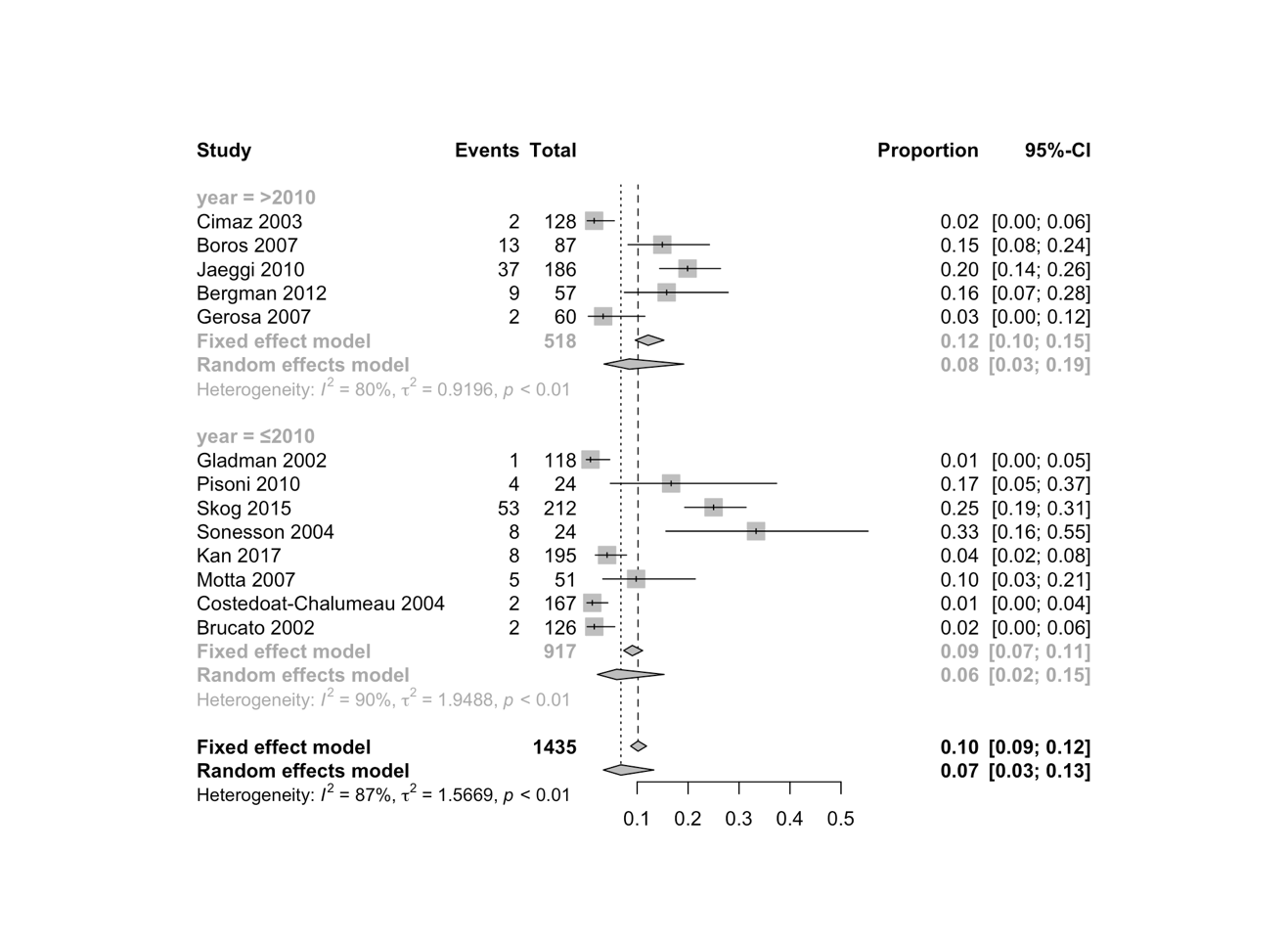


Fig.S5 sensitivity analysis


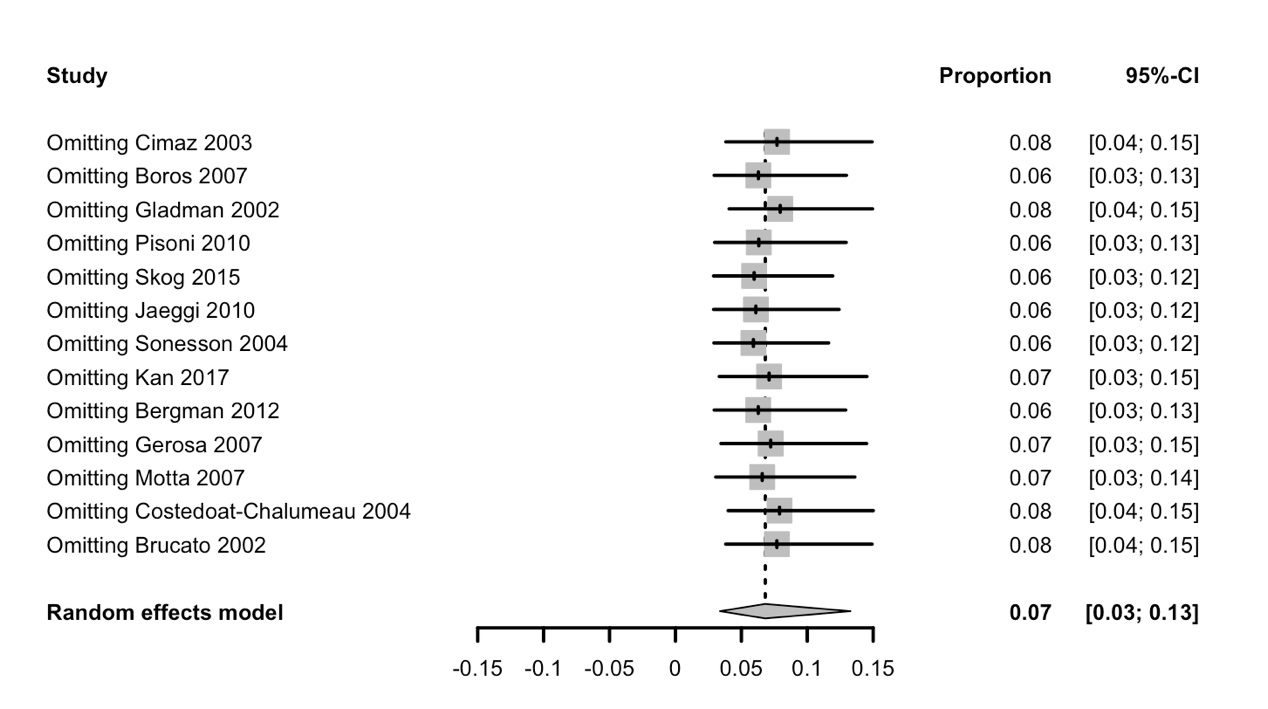

Supplement: Supplementary file 1 — Additional file 1: Fig. S1. The prevalence of CHB between SLE and non-SLE groups. Fig. S2. Subgroup analysis of different types of diagnostic method. Fig. S3. Subgroup analysis based on regions of studies. Fig. S4. Subgroup analysis based on publication time. Fig. S5. Sensitivity analysis. [file 12969_2023_803_MOESM1_ESM.docx]
